# Proximal tibiofibular synostosis in HDP SPECT/CT bone scan: A case report

**DOI:** 10.1016/j.radcr.2025.04.113

**Published:** 2025-05-21

**Authors:** Eliluane Pirazzo Andrade Teixeira, Charles-Edouard Decorads, Inès Castarède, Sana Boudabbous, Valentina Garibotto

**Affiliations:** a Department of Diagnosis, Division of Nuclear Medicine and Molecular Imaging, Geneva University Hospitals, Geneva. Switzerland; b Department of Diagnosis, Division of Radiology, Geneva University Hospitals, Geneva, Switzerland; c Faculty of Medecine, Geneva University, Geneva, Switzerland

**Keywords:** SPECT/CT, Bone scintigraphy, Synostosis, Tibiofibular joint, HDP

## Abstract

We report the HDP SPECT/CT findings in a case of proximal tibiofibular synostosis in a 47-year-old man with a history of repetitive microtraumas from former sports activities. Characterization of this rare entity using 99mTc-HDP SPECT/CT is essential for understanding its pathogenesis and guiding appropriate treatment strategies.

## Case report

A 47-year-old man, previously active in martial arts, skateboarding, and snowboarding for over 2 decades, presented with chronic right knee pain radiating from the foot to the mid-thigh. The pain was constant, aggravated by movement, and exacerbated by cold, rainy, or windy weather. He reported a progressive enlargement of a palpable bony mass at the fibular head. Physiotherapy failed to alleviate symptoms, and complex regional pain syndrome (CRPS) was initially suspected.

Initial plain radiographs and MRI of the right knee demonstrated an enlarged fibular head without signs of dislocation or subluxation. A 3-phase bone scintigraphy using technetium-99m HDP revealed focal increased radiotracer uptake at the proximal tibiofibular joint, suggestive of elevated osteoblastic activity.

Subsequent HDP SPECT/CT imaging confirmed the presence of a bony bridge between the proximal tibia and fibula, indicative of a synostosis [1]. There was no evidence of joint dislocation or subluxation. The absence of a cartilaginous cap on MRI excluded the diagnosis of osteochondroma. These findings were consistent with a nonosteochondroma-related proximal tibiofibular synostosis.

## Discussion

**Fig. 1:**
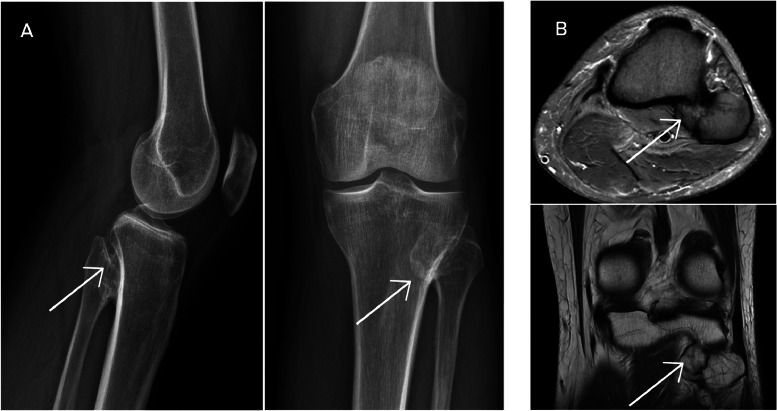
(A) Lateral, anteroposterior, and posteroanterior plain radiographs of the right knee showing an enlarged fibular head. (B) Axial and coronal MRI images confirming the enlarged fibular head without dislocation or subluxation.

**Fig. 2:**
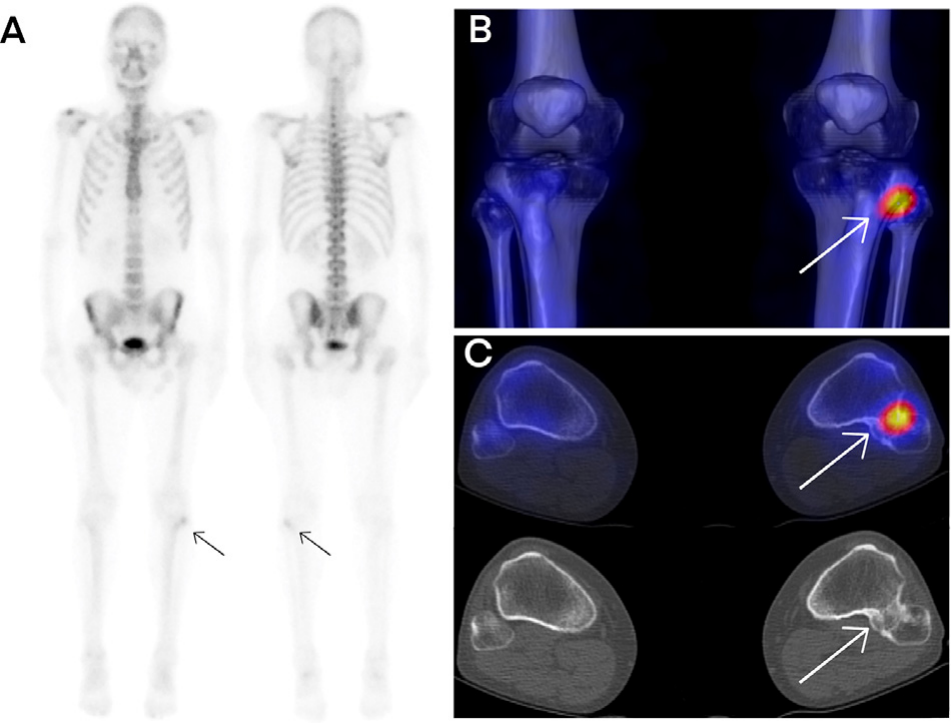
(A) Whole-body scintigraphy showing focal increased uptake at the proximal tibiofibular joint. (B) 3D SPECT/CT fusion and (C) 2D SPECT/CT images demonstrating bony bridging between the proximal tibia and fibula.

**Fig. 3:**
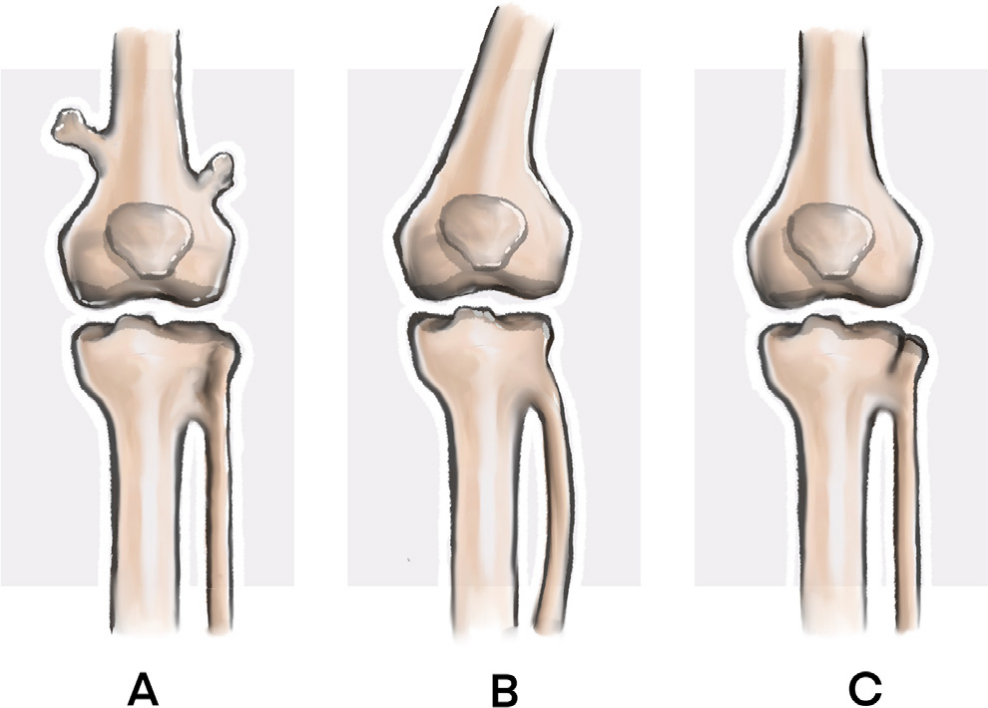
Schematic representation of Takai's classification of proximal tibiofibular synostosis: Type A (osteochondroma-related), Type B (congenital/childhood-onset), and Type C (acquired in adulthood).

Proximal tibiofibular synostosis is a rare condition that can have either congenital or acquired origins [2]. It is frequently overlooked due to its nonspecific clinical presentation, which may include posterolateral knee pain, localized swelling, and reduced ankle mobility. The rarity and subtlety of symptoms contribute to delayed diagnosis and misinterpretation (Fig. 1, Fig. 2, Fig. 3).

Radiographic evaluation typically reveals bony bridging between the proximal tibia and fibula [3]. Differentiating congenital from acquired forms—resulting from repetitive trauma, previous surgery, or inflammatory processes—is crucial for determining prognosis and management. MRI plays a key role by demonstrating cortical continuity and the absence of a cartilaginous cap, features that help rule out osteochondroma.

Takai et al. proposed a classification system for proximal tibiofibular synostosis [4]:

• ⁠Type A: Osteochondroma-related.
• Type B: Congenital or childhood-onset.
• Type C: Acquired in adulthood.

Based on the patient’s clinical history and imaging findings, this case aligns with Type C synostosis, likely acquired due to repeated microtraumas over years of intense physical activity.

Management is typically conservative in asymptomatic or mildly symptomatic cases. In cases with persistent pain or functional impairment, surgical intervention may be considered. Options include resection of the fibular head, excision of the synostosis, or corrective osteotomy.

## Conclusion

This case highlights the diagnostic utility of HDP SPECT/CT in evaluating proximal tibiofibular synostosis [5]. This imaging modality provides both anatomical and metabolic information, facilitating differentiation between congenital and acquired forms and supporting treatment planning in symptomatic patients.

## Patient consent

I confirm that written informed consent was obtained from the patient for the publication of this case report, including all accompanying images and clinical information. The patient has approved the content and understands that all identifying details will remain confidential.
